# Effects of Sodium Butyrate on Sperm Function and Protein Acetylation in Fresh and Frozen–Thawed Boar Spermatozoa

**DOI:** 10.3390/ani16131952

**Published:** 2026-06-24

**Authors:** Grzegorz Smołucha, Monika Trzcińska, Magdalena Bryła, Anna Steg, Lechosław Gajda

**Affiliations:** 1 Department of Animal Molecular Biology, Laboratory of Proteomics, National Research Institute of Animal Production, Krakowska 1 Street, 32-083 Balice, Poland; anna.steg@iz.edu.pl; 2 Department of Reproductive Biotechnology and Cryoconservation, National Research Institute of Animal Production, Krakowska 1 Street, 32-083 Balice, Poland; monika.trzcinska@iz.edu.pl (M.T.); magdalena.bryla@iz.edu.pl (M.B.); lechoslaw.gajda@iz.edu.pl (L.G.)

**Keywords:** boar sperm, sodium butyrate, cryopreservation, protein acetylation, sperm motility, mitochondrial function

## Abstract

Sodium butyrate (NaBu), a short-chain fatty acid and histone deacetylase inhibitor, has been reported to influence protein acetylation and cellular function; however, its effects on boar spermatozoa remain poorly understood. This study evaluated the effects of NaBu on sperm function and global protein acetylation in fresh after 24 h storage and frozen–thawed boar spermatozoa. Semen samples collected from boars (n = 4), with three ejaculates per boar, were supplemented with 0, 0.5, 0.75, or 1 mM NaBu, stored for 24 h at 17 °C, and subsequently cryopreserved. Sperm motility, mitochondrial membrane potential, membrane integrity, apoptosis-like changes, and chromatin status were assessed using CASA, flow cytometry, and fluorescence microscopy, whereas global protein acetylation was assessed by Western blotting. In fresh semen after 24 h storage, NaBu did not significantly affect the evaluated sperm functional parameters, whereas frozen–thawed spermatozoa showed significant changes in selected functional parameters, particularly total and progressive motility at 0.5 mM. Selected mitochondrial membrane potential parameters were also affected in frozen–thawed samples, while membrane integrity, apoptosis-like changes, and chromatin status remained largely unaffected. NaBu did not significantly alter global protein acetylation levels in either fresh after 24 h storage or frozen–thawed spermatozoa. Considerable inter-individual variability between boars was observed. These findings indicate that NaBu may affect selected in vitro functional properties of frozen–thawed boar spermatozoa; however, the observed functional changes were not associated with detectable statistically significant changes in global protein acetylation under the conditions tested. Further studies are needed to determine whether specific acetylated proteins, metabolic pathways, or stress-response mechanisms are involved.

## Simple Summary

Artificial insemination in pigs relies heavily on high-quality semen, but freezing and thawing sperm cells for long-term storage can damage spermatozoa and reduce their movement and survival. Finding safe additives that may help protect sperm during cryopreservation is therefore important for animal breeding and food production. In this study, we investigated whether sodium butyrate, a naturally occurring short-chain fatty acid known to influence protein regulation in cells, could affect the quality of boar sperm after storage and freezing. Semen samples from four boars were treated with different concentrations of sodium butyrate and analyzed for sperm motility, membrane integrity, mitochondrial activity, apoptosis-like changes, chromatin stability, and global protein acetylation. The results showed that sodium butyrate was associated with changes in selected motility and mitochondrial parameters in frozen–thawed samples. At the same time, little effect was observed in fresh semen stored for 24 h. These functional changes occurred without statistically significant detectable changes in global protein acetylation under the conditions tested; however, this does not exclude the involvement of specific acetylated proteins or other molecular mechanisms. Considerable differences were also observed between individual boars, indicating inter-individual variability in response to sodium butyrate treatment. These findings may support further research aimed at improving boar semen preservation methods, but additional studies, including fertility-related outcomes, are needed before practical application can be recommended.

## 1. Introduction

Post-translational modifications (PTMs) expand protein functional diversity beyond genetic control. Among these, lysine acetylation, set by histone acetyltransferases and reversed by histone deacetylases, modulates protein structure, interactions, and activity, thereby influencing chromatin remodeling, metabolism, and signaling. In spermatozoa, acetylation is increasingly recognized as a regulator of maturation, capacitation, motility, and fertilization competence, with multiple studies showing acetylated proteins across the flagellum, mitochondria, and plasma membrane as well as dynamic changes during capacitation [1,2,3]. Sodium butyrate (NaBu), a short-chain fatty acid, is a histone deacetylase inhibitor (HDACi) that may influence protein acetylation by inhibiting class I/II HDAC activity. Although its genome-level effects are well described in somatic cells, mature spermatozoa are largely transcriptionally and translationally silent. Therefore, short-term exposure to NaBu is unlikely to act primarily through de novo transcriptional or translational responses. In mature spermatozoa, NaBu-related effects may instead involve post-translational regulation of pre-existing proteins, including changes in protein acetylation status [4,5]. Studies in mouse and human spermatozoa have suggested that acetylation-related mechanisms may be involved in the regulation of sperm function, including motility, capacitation, and fertilization competence. Though these studies were performed in different species and under different experimental conditions, together they indicate that acetylation-related mechanisms may be relevant to sperm function across mammalian models and provide a rationale for investigating this process in boar spermatozoa [2,3,6]. In pig sperm, available evidence remains limited; however, acetylation-related changes have been associated with motility regulation, acrosome status, capacitation-related events, and semen processing, including cryopreservation. In boar spermatozoa, Chen et al. reported that lysine acetylation may participate in the regulation of motility and acrosome integrity under different glucose conditions [3]. More recently, Ali et al. showed that cryopreservation induced acetylation and deacetylation changes in energy metabolism-related proteins in boar sperm, which may contribute to post-thaw sperm quality [5]. However, these studies do not establish a universal quantitative relationship or direct causality between global protein acetylation and sperm motility. Therefore, acetylation should be considered a potential molecular feature associated with sperm functional status rather than a proven causal determinant of motility [7,8,9]. Finally, during spermatogenesis, histone acetylation is integral to histone-to-protamine exchange and nuclear remodeling, leaving a small, regulatory fraction of histones in mature sperm that may carry epigenetic information. This further underscores why acetylation pathways remain relevant from spermiogenesis into sperm function [10,11,12]. Polish Landrace is an important maternal pig breed widely used in commercial pig production and breeding programs in Poland. Therefore, improving semen preservation in this breed has practical relevance for artificial insemination efficiency, genetic dissemination, and the economic sustainability of pig production [13]. Based on the known biological activity of sodium butyrate, including its reported effects on protein acetylation and cellular metabolism, we hypothesized that NaBu supplementation would improve selected functional parameters of boar spermatozoa, particularly under cryopreservation-induced stress. We further hypothesized that these functional changes may be accompanied by detectable alterations in global protein acetylation. Therefore, the aim of this study was to evaluate the effect of sodium butyrate on global protein acetylation and sperm functional parameters in fresh after 24 h storage and frozen–thawed boar semen, and to determine whether changes in global acetylation are associated with sperm quality.

## 2. Materials and Methods

### 2.1. Experimental Animals

Semen samples were obtained from four Polish Landrace boars aged 2–3 years. Three ejaculates were collected from each boar at different time points, resulting in 12 biological samples. Because repeated ejaculates were obtained from the same boars, these samples were treated as biological samples nested within boar rather than as fully independent animal-level replicates. Each ejaculate was divided into four experimental groups supplemented with 0, 0.5, 0.75, or 1 mM sodium butyrate (NaBu). For clarity, the treatment groups are referred to throughout the manuscript according to the NaBu concentration used: 0 mM, 0.5 mM, 0.75 mM, and 1.0 mM, with 0 mM representing the untreated control group. All treatment groups were subsequently evaluated under both fresh (after 24 h storage at 17 °C) and cryopreserved conditions. Multiple technical and analytical measurements were performed for each experimental condition. Animals were housed at the Boar AI Station in Czermin under controlled environmental conditions (temperature and humidity). Animals were fed a commercial complete diet formulated for breeding boars (Knur Livelle Optima CT549X0T, Cargill Poland Sp. z o.o., Warsaw, Poland) at 2.3 kg/day, with water available ad libitum. Detailed diet composition is provided in Supplementary Table S6. Ethical review and approval were waived because semen collection was performed as part of routine breeding and artificial insemination procedures, without any experimental intervention or additional handling of animals beyond standard husbandry practices. This was confirmed by the official opinion issued by the Animal Welfare Team Leader of the Local Ethics Committee (Supplementary Materials).

### 2.2. Semen Collection and Preparation

The sperm-rich fraction was collected by the gloved-hand technique into pre-warmed water-jacketed vessels. Immediately after collection, semen was diluted at a 1:1 ratio using Biosolwens Plus extender (Biochefa, Sosnowiec, Poland) and transported to the laboratory. Each diluted sperm-rich fraction was divided into four treatment groups: 0 mM (control) without sodium butyrate (NaBu) and with 0.5, 0.75, and 1.0 mM NaBu. Sodium butyrate (≥98% purity, Sigma-Aldrich, St. Louis, MO, USA) was used in the study. The pH and osmolarity of the NaBu-supplemented extender ranged from 7.2 to 7.4 and from 290 to 320 mOsm/kg, respectively. All samples were stored for 24 h at 17 °C and subsequently cryopreserved according to a patented method (no. PL 228192). Each ejaculate was analyzed separately, without pooling, and was considered a biological semen sample nested within boar.

### 2.3. Cryopreservation

Spermatozoa were cryopreserved using a patented method (Patent No. PL 228192). Diluted semen was transferred into 50-mL centrifuge tubes and centrifuged at 800× *g* for 25 min at 17 °C. The supernatant was discarded, and the sperm pellet was resuspended in LEY extender (80 mL of 11% lactose solution and 20 mL of egg yolk) to a final concentration of 1.5 × 10^9^ spermatozoa/mL. The samples were maintained at 17 °C and equilibrated for 120 min. Subsequently, two parts of semen were mixed with one part of LEYG extender consisting of 89.5% LEY extender, 9% glycerol, and 1.5% Equex-STM paste (Nova Chemical Sales, Scituate, MA, USA), whose active ingredient is sodium dodecyl sulfate. Cryopreservation was performed using a manual freezing protocol. The cooled and diluted semen was packaged into 0.5-mL straws and placed on a rack positioned 3 cm above the surface of liquid nitrogen (−120 °C) for 20 min inside a polystyrene box. Subsequently, the straws were plunged into liquid nitrogen (−196 °C) and stored in cryogenic containers until analysis. For thawing, the straws were immersed in a circulating water bath at 37 °C for 40 s. Immediately after thawing, semen was diluted in BP extender pre-warmed to 37 °C and incubated for 15 min at 37 °C prior to post-thaw evaluation.

### 2.4. Semen Evaluation

All semen samples, liquid-preserved for 24 h at 17 °C and cryopreserved, were evaluated using the following methods.

### 2.5. Assessment of Sperm Motility

Sperm motility was evaluated using a computer-assisted sperm analysis system (CASA; Sperm Class Analyzer, SCA^®^ version 5.1, Microptic S.L., Barcelona, Spain). Prior to analysis, semen samples were incubated at 38 °C for 15 min. A 2-µL aliquot of semen was loaded into a 20-µm-deep Leja counting chamber (Leja Products B.V., Nieuw-Vennep, The Netherlands) and examined under a phase-contrast microscope equipped with a 10× objective and a Basler ACA1300-200UC digital camera (Basler AG, Ahrensburg, Germany). Image acquisition was performed at 25 frames/s. For each replicate, five microscopic fields were analyzed and at least 1000 spermatozoa were evaluated. CASA settings were as follows: sperm head area 10–80 µm^2^, static spermatozoa < 10 µm/s, slow motile spermatozoa 10–25 µm/s, medium motile spermatozoa 25–45 µm/s, rapid motile spermatozoa > 45 µm/s, connection distance 11 µm, VAP threshold 5 µm/s, and progressive motility threshold according to the predefined SCA settings (STR ≥ 45%). Only total motility (TMOT) and progressive motility (PMOT) were recorded for further analysis.

### 2.6. Flow Cytometry Analysis

Flow cytometry was performed using a CytoFLEX cytometer (Beckman Coulter, Inc., Brea, CA, USA) equipped with CytExpert software, version 2.4.0.28 (Beckman Coulter, Inc.).

### 2.7. Sperm Chromatin Structure Assay (SCSA)

The sperm chromatin structure assay (SCSA) was used to assess sperm chromatin integrity. DNA fragmentation was expressed as the DNA fragmentation index (DFI), representing the percentage of spermatozoa with fragmented DNA. Semen samples were placed on crushed ice and protected from light. An aliquot of 100 µL of semen (1 × 10^6^ sperm/mL) was mixed with 200 µL of a permeabilizing solution containing 1 N HCl (POCH, Gliwice, Poland), Triton X-100 (Sigma-Aldrich, St. Louis, MO, USA), and NaCl (Sigma-Aldrich, St. Louis, MO, USA) (8 mL 1 N HCl, 0.1 mL Triton X-100, 0.877 g NaCl, distilled water up to 100 mL) for 30 s to induce DNA denaturation. Subsequently, 600 µL of staining solution containing 37 mL of 0.1 M citric acid (Sigma-Aldrich, St. Louis, MO, USA), 63 mL of 0.2 M Na2HPO4 (Sigma-Aldrich, St. Louis, MO, USA), 0.877 g NaCl (Sigma-Aldrich, St. Louis, MO, USA), 34 mg EDTA (Sigma-Aldrich, St. Louis, MO, USA), and 0.6 mg acridine orange (Sigma-Aldrich, St. Louis, MO, USA) was added. Samples were incubated for 3 min and analyzed within 30 min. Following excitation with a 488-nm blue laser, acridine orange bound to double-stranded DNA emitted green fluorescence (525 nm; 525/40 BP detector), whereas acridine orange associated with denatured or single-stranded DNA emitted red fluorescence (690 nm; 690/50 BP detector). Debris was excluded based on forward scatter (FSC) and side scatter (SSC) characteristics, and sperm populations were further gated using red versus green fluorescence cytograms. The DNA fragmentation index was calculated as DFI (%) = [red fluorescence/(red fluorescence + green fluorescence)] × 100. Data acquisition was performed using a CytoFLEX flow cytometer (Beckman Coulter, Brea, CA, USA) and CytExpert software version 2.4.0.28 (Beckman Coulter). Data were analyzed using WinList 3D software (version 9.0.1). A total of 10,000 spermatozoa were acquired and analyzed for each sample. To ensure consistency of instrument settings between measurements, a reference boar semen sample was included in each analytical session.

### 2.8. Assessment of Plasma Membrane Integrity (SYBR-14/PI Staining)

Plasma membrane integrity was evaluated using a commercial viability kit (LIVE/DEAD Sperm Viability Kit, Invitrogen, L7011, Eugene, OR, USA). Semen samples were adjusted to a concentration of 5 × 10^6^ spermatozoa/mL in a final volume of 1 mL. For staining, 5 µL of the working solution (prepared by diluting 1 µL SYBR-14 in 49 µL distilled H_2_O) was added to each sample, followed by incubation for 10 min at 37 °C in the dark. Subsequently, 2 µL of propidium iodide (PI) solution (2 µg/mL) was added, and samples were incubated for an additional 2 min prior to analysis. SYBR-14, a membrane-permeant nucleic acid dye, labels viable spermatozoa with intact membranes, producing bright green fluorescence (emission detected at 525 nm; 525/40 BP filter). PI, a classical membrane-impermeant viability marker, penetrates only membrane-compromised cells, resulting in red fluorescence (emission detected at 690 nm; 690/50 BP filter). Initial gating to exclude debris and non-sperm particles was performed using forward scatter (FSC) versus side scatter (SSC) dot plots. A total of 10,000 spermatozoa were analyzed for each sample. Events within the gated sperm population were subsequently classified based on SYBR-14 and PI fluorescence profiles: viable (live) spermatozoa—intact membranes; SYBR-14^+^/PI^−^ (green fluorescence), nonviable (dead) spermatozoa—membrane-damaged; SYBR-14^−^/PI^+^ (red fluorescence), membrane-compromised (dying) spermatozoa—transitional or partially damaged membranes; SYBR-14^+^/PI^+^ (green and red fluorescence).

### 2.9. Fluorescence Microscopy Assessment of Semen

Semen smears were prepared and examined using a fluorescence microscope (Nikon Eclipse E600, Tokyo, Japan).

### 2.10. Detection of Apoptotic-like Changes in Spermatozoa (YO-PRO-1/PI Assay)

Apoptotic-like alterations in sperm plasma membrane permeability were assessed using the Vybrant Apoptosis Assay Kit #4 (Molecular Probes Inc., Eugene, OR, USA). Semen samples were diluted in 1 mL of phosphate-buffered saline (PBS; Sigma-Aldrich Chemie GmbH, Steinheim, Germany). Subsequently, 2 μL of YO-PRO-1 (100 μmol/L) was added to each sample. The tubes were gently mixed and incubated for 20 min at room temperature in the dark. Following this incubation, PI was added to a final concentration of 1 μmol/L, and samples were incubated for an additional period prior to evaluation. Spermatozoa were examined using a fluorescence microscope equipped with filter sets appropriate for YO-PRO-1 (Ex 491 nm/Em 507 nm) and PI (Ex 538 nm/Em 619 nm). For each sample, a minimum of 200 spermatozoa were assessed in a single field by the same observer to avoid inter-observer variability. Spermatozoa were classified into the following categories: viable spermatozoa (YO-PRO-1^−^/PI^−^), viable spermatozoa with apoptotic-like changes (YO-PRO-1^+^/PI^−^) and nonviable spermatozoa (YO-PRO-1^+^/PI^+^).

### 2.11. Assessment of Mitochondrial Membrane Potential Using the JC-1 Probe

Mitochondrial membrane potential (ΔΨm) was evaluated using the cationic fluorescent probe JC-1 (Molecular Probes Inc., Eugene, OR, USA). Semen samples were centrifuged at 300× *g* for 15 min at room temperature, and the resulting pellet was washed twice with calcium- and magnesium-free phosphate-buffered saline (PBS; Sigma-Aldrich Chemie GmbH, Steinheim, Germany). Following the final wash, the sperm pellet was resuspended in 1 mL of PBS. Samples were stained with JC-1 at a final concentration of 3 µg/mL (from a stock solution of 1 mg/mL prepared in DMSO) and incubated for 15 min at 37 °C in the dark. For each sample, a minimum of 200 spermatozoa were evaluated in a single field by the same observer to minimize inter-observer variability.

JC-1 staining differentiated spermatozoa based on mitochondrial functionality: in spermatozoa with high ΔΨm, JC-1 selectively accumulates within the mitochondria, forming J-aggregates that emit red to orange fluorescence; in spermatozoa with low ΔΨm, due to impaired mitochondrial membrane potential, JC-1 remains in its monomeric form within the cytoplasm and exhibits green fluorescence.

### 2.12. Protein Extraction

Sperm pellets were lysed in Lysis buffer (7 M Urea, 2 M thiourea, 2% (*w*/*v*) CHAPS, 50 mM DTT, protease and deacetylase inhibitor cocktail—ThermoFisher, Waltham, MA, USA). Lysates were sonicated (20 s sonication, 30 s rest) × 15 cycles (Bioruptor, Diagenode, Seraing, Belgium) and centrifuged at 14,000× *g* for 15 min at 4 °C. Supernatants were collected, and total protein concentration was determined by the Bradford assay (Bio-Rad, Hercules, CA, USA).

### 2.13. SDS-PAGE and Western Blot

Equal amounts of protein (20 µg per lane) were mixed with Laemmli sample buffer and denatured at 95 °C for 5 min. Samples were separated by SDS-PAGE (12% polyacrylamide gel) and transferred onto PVDF membranes (0.45 µm, pre-activated in methanol) using a semi-wet transfer system (Bio-Rad). Membranes were blocked with 5% BSA in TBS-T (Tris-buffered saline with 0.1% Tween-20) for 1 h at room temperature and then incubated overnight at 4 °C with primary antibodies: anti-acetyl-lysine antibody (Cell Signaling Technology, Danvers, MA, USA, Cat. No. 9441, RRID: AB_331805; 1:1000) and anti-β-tubulin antibody (Thermo Fisher Scientific, Waltham, MA, USA, Cat. No. PA1-16947, RRID: AB_795659; 1:5000). After washing, membranes were incubated with goat anti-rabbit HRP-conjugated secondary antibody (Thermo Fisher Scientific, Cat. No. 31460, RRID: AB_228341; 1:20,000) for 1 h at room temperature. Bands were visualized using ECL substrate and imaged with a chemiluminescence detection system (ChemiDoc, Bio-Rad). Chemiluminescent signals were acquired using the automatic exposure mode of the ChemiDoc system within the non-saturated linear detection range. Densitometric analysis was performed using Image Lab software Version 6.0.1 (Bio-Rad), and signal intensities were normalized to beta-tubulin.

### 2.14. Statistical Analysis

Data were analyzed using linear mixed-effects models (LMMs) implemented in the lme4 package (Version 2.0-1) in RStudio 2024.04.2, with *p*-values obtained using lmerTest. In the main models, sperm functional parameters were used as dependent variables, while NaBu dose, acetylation level, and sample condition (fresh after 24 h storage vs. frozen–thawed) were included as fixed effects. To account for repeated measurements and inter-individual variability, boar and collection nested within boar were included as random effects. For each linear mixed-effects model, marginal R^2^ and conditional R^2^ were calculated to estimate the variance explained by fixed effects alone and by the full model including both fixed and random effects, respectively.

To determine whether the effect of NaBu dose differed between fresh after 24 h storage and frozen–thawed spermatozoa, additional global LMMs including the dose × condition interaction were fitted for each sperm functional parameter. In these models, dose, condition, and the dose × condition interaction were treated as fixed effects, whereas boar and collection nested within boar were included as random effects:

Y ∼ dose × condition + (1 | boar) + (1 | boar:collection)

For condition-specific analyses, fresh after 24 h storage and frozen–thawed samples were analyzed separately. In these models, dose was treated as a categorical fixed effect and tested using LMMs of the form:

Y ∼ dose + (1 | boar) + (1 | boar:collection)

The global effect of dose was assessed using Type III ANOVA F-tests. When significant, post hoc pairwise comparisons between dose levels were performed using estimated marginal means with Tukey adjustment. For relevant post hoc comparisons, effect estimates, standard errors, 95% confidence intervals, and adjusted *p*-values were reported.

The effect of NaBu on global acetylation was analyzed using LMMs with dose as a fixed effect and boar as a random effect:

acetylation ∼ dose + (1 | boar)

Additionally, for the analysis of global protein acetylation, densitometric values of acetyl-lysine signals were first normalized to the corresponding β-tubulin signal to account for loading differences between lanes. To evaluate treatment-related changes, normalized acetylation values were then expressed relative to the corresponding control group (0 mM NaBu) within each boar and sample condition. These relative values were log10-transformed before statistical analysis to reduce skewness and improve the distribution of residuals. Statistical comparisons were performed on the log10-transformed values, whereas non-transformed or back-transformed descriptive values are presented where appropriate for biological interpretation.

*p*-values from multiple parameter testing were adjusted using the Benjamini–Hochberg false discovery rate (FDR) procedure. Statistical significance was set at *p* < 0.05. No a priori power analysis was performed; therefore, the limited number of boars and the statistical power to detect small treatment effects or dose × condition interactions are acknowledged as limitations of the study.

In addition to inferential statistics, descriptive summaries were calculated as mean ± SD for each boar, dose, and condition. Individual response scores were also calculated by expressing the change in selected functional parameters relative to the control dose for each boar, thereby allowing ranking of boars by their overall response to NaBu treatment.

Principal component analysis (PCA) was used as an exploratory multivariate approach to visualize clustering patterns in sperm functional parameters. PCA was performed both globally and for selected parameter groups, separately for fresh after 24 h storage and frozen–thawed samples. These analyses were used only for descriptive visualization of clustering by condition, dose, or functional parameter profile.

## 3. Results

### 3.1. Effect of NaBu on Sperm Functional Parameters in Fresh and Frozen–Thawed Samples

**Figure 1:**
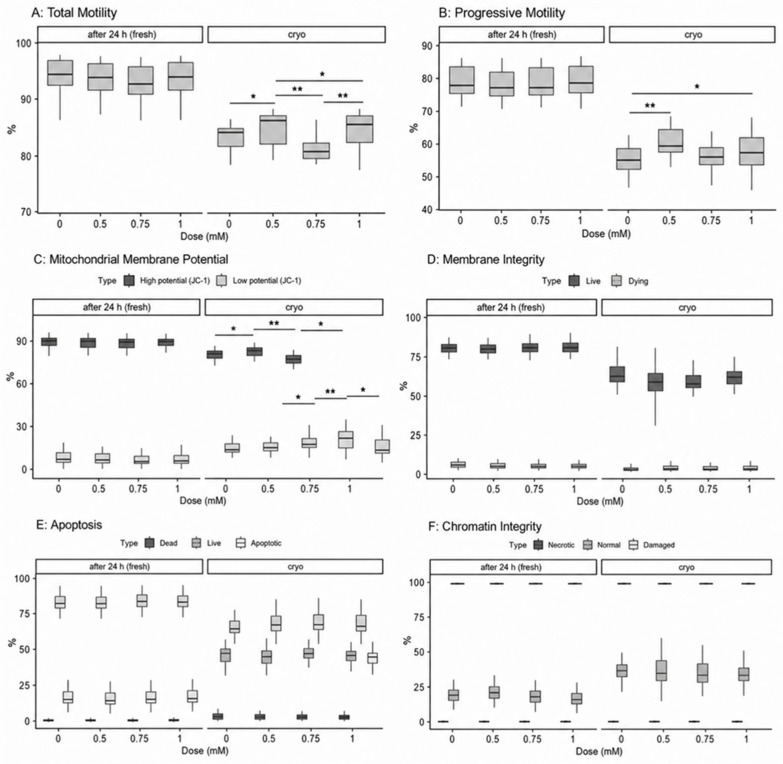
Effect of sodium butyrate (NaBu; 0–1 mM) on functional parameters of boar sperm under fresh after 24 h storage and frozen–thawed conditions. Data are presented as boxplots showing median, interquartile range, and whiskers. Semen samples were obtained from four boars, with three ejaculates per boar, resulting in 12 biological samples. Each ejaculate was divided into four NaBu treatment groups. Statistical significance was assessed using linear mixed-effects models with dose as a fixed effect and boar and collection nested within boar as random effects, followed by Tukey-adjusted post hoc comparisons. Significant differences between doses are indicated as follows: * *p* < 0.05, ** *p* < 0.01.

In fresh semen after 24 h storage, total motility (TM) and progressive motility (PM) did not differ significantly among NaBu treatment groups (*p* > 0.05; Figure 1A,B; Supplementary Table S1). No significant differences among NaBu treatment groups were detected for mitochondrial membrane potential, membrane integrity, apoptosis-like changes, or chromatin status in fresh samples (*p* > 0.05; Figure 1C–F; Supplementary Table S1).

In frozen–thawed spermatozoa, significant differences among NaBu treatment groups were observed for selected parameters, including total motility, progressive motility, and mitochondrial membrane potential (Figure 1A–C; Table 1 and Supplementary Table S2). Membrane integrity, apoptosis-like changes, and chromatin status did not differ significantly among NaBu treatment groups (Figure 1D–F; Supplementary Table S2). For clarity, Table 1 presents the main post-thaw functional parameters, whereas the complete statistical output for all evaluated sperm parameters, including non-significant comparisons, is provided in Supplementary Table S2.

To formally test whether the effect of NaBu dose differed between fresh after 24 h storage and frozen–thawed spermatozoa, global mixed-effects models including the dose × condition interaction were fitted for each sperm functional parameter. After FDR correction, the dose × condition interaction was not statistically significant for any evaluated parameter (Supplementary Table S4). Therefore, the dose-related differences observed mainly in frozen–thawed samples are presented as condition-specific findings rather than as definitive evidence of a statistically confirmed dose × condition interaction.

Pairwise comparisons within each condition from the global interaction models are provided in Supplementary Table S5. These comparisons confirmed the absence of significant dose-related differences in fresh samples and revealed selected dose-related differences in frozen–thawed samples, primarily in motility and mitochondrial membrane potential.

### 3.2. Effect of NaBu on Global Protein Acetylation

**Figure 2:**
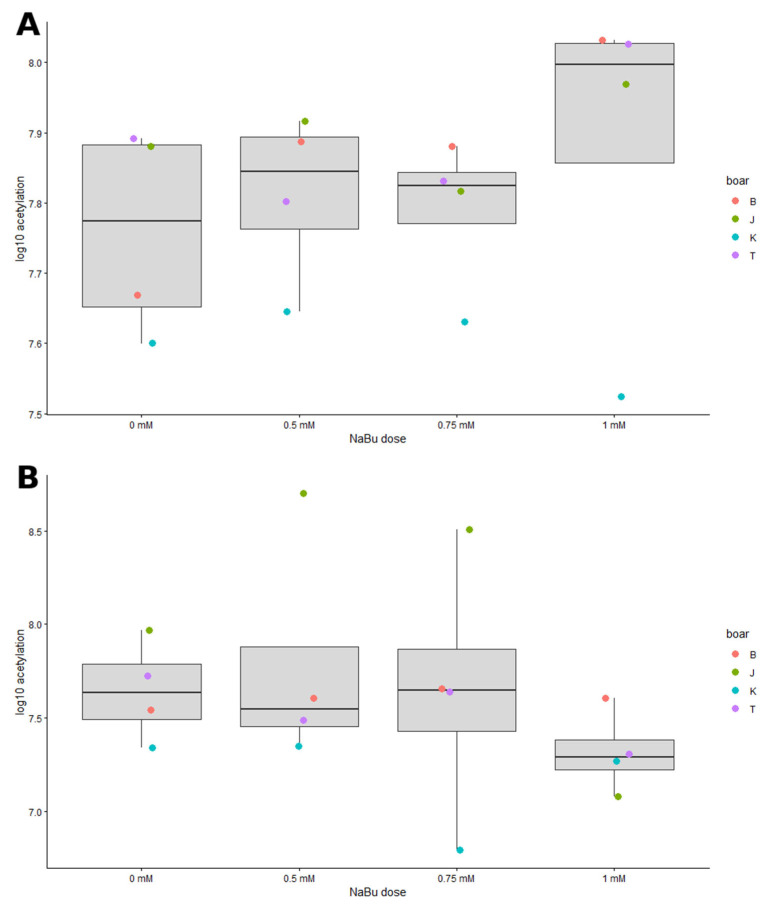
Log10-transformed acetylation levels in boar sperm treated with sodium butyrate (NaBu; 0–1 mM). (**A**) Frozen–thawed. (**B**) Fresh sperm after 24 h storage. Data are presented as boxplots, with individual data points representing each boar. Letters B, J, K, and T indicate anonymized identifiers of individual boars and do not represent treatment groups.

NaBu did not significantly alter global acetylation levels in either fresh after 24 h storage or frozen–thawed spermatozoa (all adjusted *p*-values > 0.05; Figure 2; Table S3). Representative Western blot images are provided in Supplementary Figure S1.

**Figure 3:**
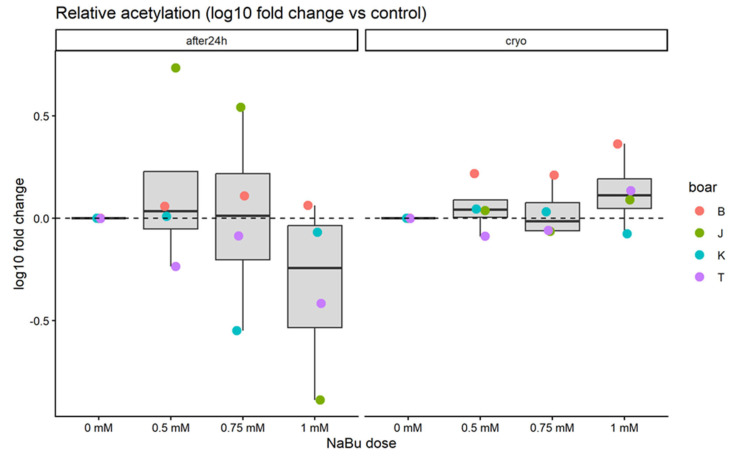
Relative acetylation levels in boar sperm following sodium butyrate (NaBu; 0–1 mM) treatment, expressed as log_10_ fold change relative to control (0 mM). Acetylation responses are shown separately for fresh sperm after 24 h storage (**left panel**) and frozen–thawed sperm (**right panel**). Data are presented as boxplots (median, interquartile range) with individual data points representing individual boars. No statistically significant differences in relative acetylation were observed between NaBu doses in either condition (all adjusted *p*-values > 0.05), although substantial inter-individual variability was evident. Letters B, J, K, and T indicate anonymized identifiers of individual boars and do not represent treatment groups.

Although acetylation values showed visible dispersion between individual boars and treatment groups, no statistically significant differences were detected between NaBu doses in either condition. Similar results were obtained when acetylation was expressed as log10-transformed fold change relative to the control (Figure 3). These findings indicate that NaBu treatment was not associated with detectable changes in global protein acetylation under the conditions tested (Figure 3).

Mean acetylation values varied between doses and individual boars, but these differences were not statistically significant (Figure 2B; Table 2).

### 3.3. Global Acetylation and Sperm Functional Parameters

In linear mixed-effects models including NaBu dose, acetylation level, and sample condition as fixed effects, no significant association was observed between global acetylation and any sperm functional parameter after accounting for boar-to-boar variation and repeated collections (Table S3). For example, acetylation was not associated with total motility (estimate = −0.222, *p* = 0.575), progressive motility (estimate = 0.154, *p* = 0.738), mitochondrial membrane potential (JC-1 high: estimate = −0.161, *p* = 0.693), membrane integrity (live: estimate = −0.287, *p* = 0.781), apoptosis-like changes (dead: estimate = 0.245, *p* = 0.807), or chromatin status (normal: estimate = 0.003, *p* = 0.953). Marginal R^2^ values ranged from 0.081 to 0.837, whereas conditional R^2^ values ranged from 0.277 to 0.888, indicating that a substantial proportion of the explained variance was attributable to the full model structure, including random effects. These findings indicate that, under the conditions tested, the functional changes observed in frozen–thawed samples were not statistically associated with global acetylation levels.

### 3.4. Inter-Individual Variability in Response to NaBu

**Figure 4:**
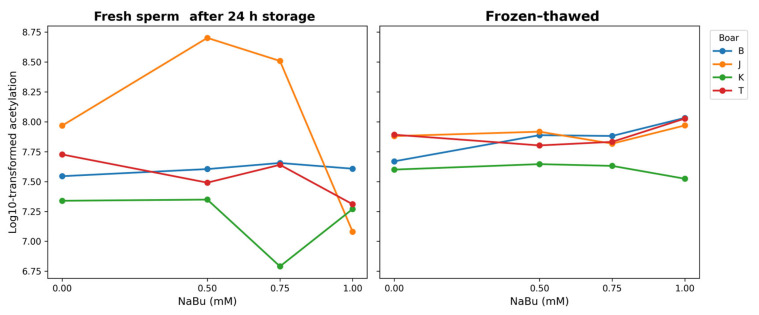
Inter-individual variability in acetylation response to sodium butyrate (NaBu) in fresh after 24 h storage and frozen–thawed boar sperm. Log10-transformed acetylation levels are shown separately for fresh sperm after 24 h storage (**left panel**) and frozen–thawed sperm (**right panel**). Lines represent mean acetylation values for individual boars across increasing NaBu concentrations (0–1 mM). Letters B, J, K, and T indicate anonymized identifiers of individual boars and do not represent treatment groups.

Substantial variability between individual boars was observed across acetylation measurements (Figure 3). Individual-level analysis revealed heterogeneous responses to NaBu. Some boars (e.g., B and J) exhibited a dose-dependent increase in acetylation, whereas others (e.g., K) showed minimal or inconsistent responses (Figure 4).

This variability was particularly evident in relative acetylation values (Figure 3) and in individual response trajectories (Figure 4), where responses diverged strongly at higher doses (0.75–1 mM).

### 3.5. Principal Component Analysis (PCA) Visualization of Sperm Functional Profiles

**Figure 5:**
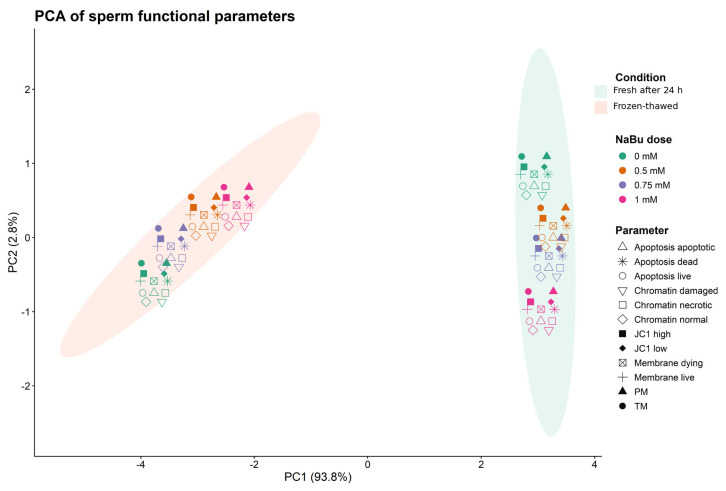
Principal component analysis (PCA) of sperm functional parameters.

PCA was used as a visualization tool and showed a separation of samples primarily according to experimental condition (fresh after 24 h storage vs. frozen–thawed) (Figure 5).

Samples are colored by NaBu dose and grouped by condition (fresh after 24 h storage vs. froze–thawed).

**Figure 6:**
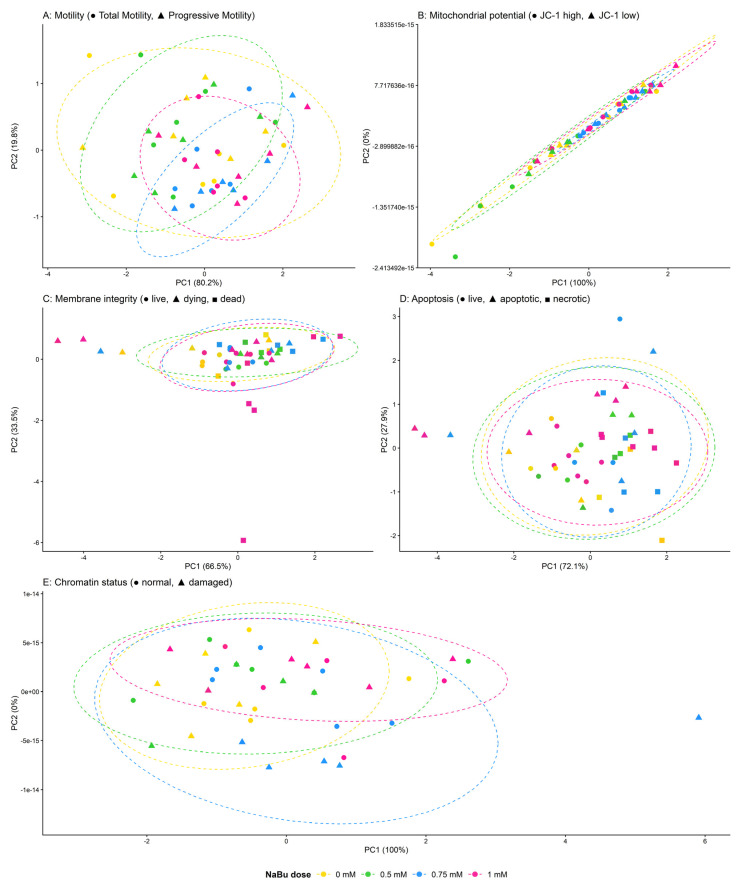
Principal component analysis (PCA) of sperm functional parameters in frozen–thawed samples treated with sodium butyrate (NaBu; 0–1 mM).

**Figure 7:**
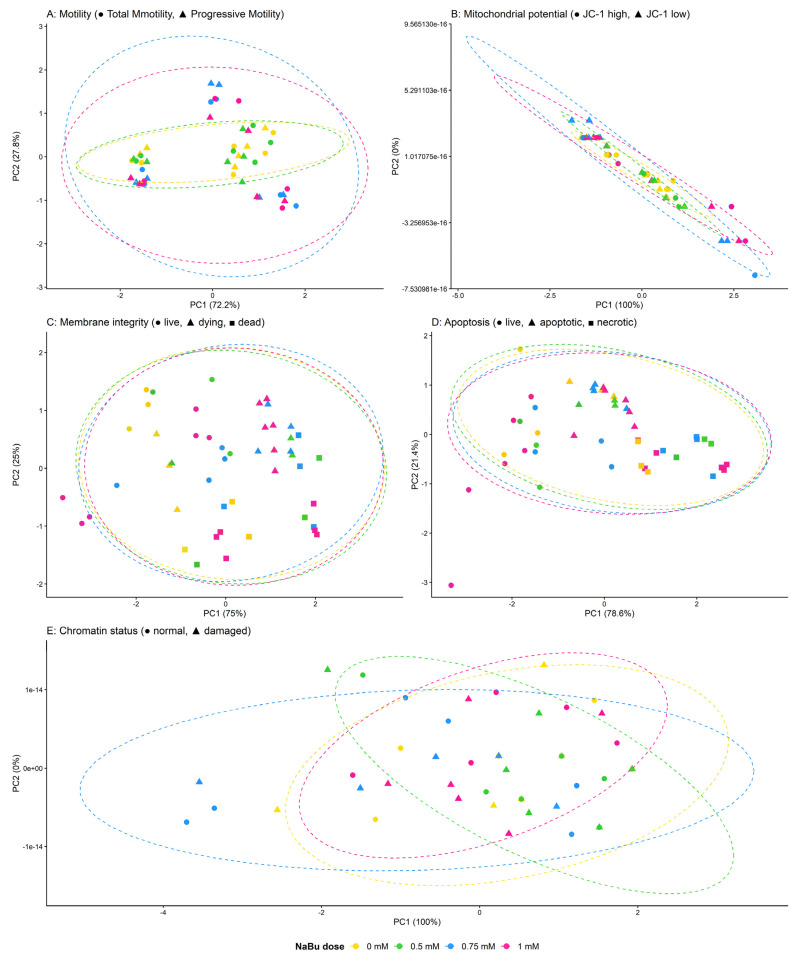
Principal component analysis (PCA) of sperm functional parameters in fresh samples treated with sodium butyrate (NaBu; 0–1 mM).

Within each condition, the NaBu dose contributed to additional variation, although the dose groups showed partial overlap (Figure 6 and Figure 7). Functional parameters such as motility, mitochondrial activity, and membrane integrity contributed to shared clustering patterns.

## 4. Discussion

The present study showed that sodium butyrate (NaBu) was associated with changes in selected functional parameters of frozen–thawed boar spermatozoa, while no corresponding significant effects were observed in fresh semen after 24 h storage. These functional changes were not associated with detectable statistically significant changes in global protein acetylation under the conditions tested.

### 4.1. NaBu-Associated Changes in Sperm Function Under Cryopreservation-Induced Stress

Cryopreservation is known to induce extensive structural and functional damage in spermatozoa, including decreased motility, mitochondrial dysfunction, and compromised plasma membrane integrity. Zhang et al. (2021) demonstrated that freeze–thawing of boar sperm leads to excessive generation of reactive oxygen species (ROS), which in turn impairs mitochondrial activity and reduces motility [14]. Similarly, Jovičić et al. (2020) reported that boar spermatozoa are particularly susceptible to cold shock due to their membrane lipid composition, which predisposes them to membrane destabilization during freezing and thawing [15]. These observations are consistent with the present findings, in which cryopreserved sperm showed reduced baseline functionality but also clear responsiveness to NaBu treatment. In contrast, fresh semen—characterized by relatively stable membrane integrity and mitochondrial function—did not respond significantly to NaBu. This observation suggests that NaBu did not measurably affect basal sperm performance under the present storage conditions, whereas its effects may become more apparent after cryopreservation-induced stress.

Comparable condition-dependent effects have been reported in studies on antioxidant supplementation. For example, Shi et al. (2022) showed that addition of mitoquinone (MitoQ) during boar semen cryopreservation improved mitochondrial function and motility, specifically in frozen–thawed samples [16]. Likewise, Yáñez-Ortiz et al. (2022) highlighted that many protective additives exert measurable benefits only in the context of cryo-induced damage [17]. These findings support the possibility that NaBu-related effects may be condition-dependent; however, the underlying mechanisms require further investigation.

### 4.2. Lack of Detectable Changes in Global Acetylation Despite NaBu Treatment

Despite its well-established role as an HDAC inhibitor, NaBu did not significantly alter global lysine acetylation levels in either fresh or cryopreserved sperm. This finding contrasts with previous studies demonstrating dynamic changes in sperm acetylation during physiological processes. Ritagliati et al. (2018) showed that lysine acetylation increases during mouse sperm capacitation and is associated with enhanced motility and signaling events [2]. Similarly, Bowker et al. (2022) reported that protein acetylation protects sperm from spontaneous acrosome reaction, indicating a functional role for acetylation in maintaining sperm stability [4]. However, it is important to note that these studies investigated dynamic processes such as capacitation, where signaling pathways are actively engaged. In contrast, the present study examined relatively stable conditions (storage and cryopreservation), where acetylation dynamics may be less pronounced or occur at a more localized level. Proteomic analyses further support this interpretation. Yu et al. (2015), using acetylproteomics in human sperm, identified hundreds of acetylated proteins involved in metabolism and motility, but these modifications were protein-specific rather than global [1]. Similarly, Ali et al. (2023) demonstrated that cryopreservation alters the acetylation status of metabolism-related proteins in boar sperm, yet these changes were detected using targeted proteomic approaches rather than global assays [5]. Therefore, the lack of detectable changes in global acetylation in the present study does not exclude the possibility of biologically relevant, protein-specific modifications that remain below the detection threshold of Western blot analysis.

### 4.3. Functional Effects of NaBu May Involve Mitochondrial and Metabolic Pathways

The absence of a direct association between global acetylation levels and sperm function suggests that NaBu may act through alternative mechanisms. Short-chain fatty acids such as butyrate are known to influence cellular metabolism and mitochondrial activity beyond their role as HDAC inhibitors. Berni Canani et al. (2012) demonstrated that butyrate regulates energy metabolism and mitochondrial function in various cell types [18], while Davie (2003) confirmed its role as a classical HDAC inhibitor [19]. In the context of spermatozoa, mitochondrial function is a key determinant of motility. Shi et al. (2022) showed that improving mitochondrial efficiency through antioxidant supplementation leads to enhanced motility in cryopreserved boar sperm [16]. In the present study, NaBu induced modest changes in mitochondrial membrane potential in cryopreserved samples, which may partially explain the observed improvements in motility. Furthermore, Zhang et al. (2021) linked cryopreservation-induced ROS production to mitochondrial dysfunction and motility loss [14]. Because ROS levels were not measured in the present study, oxidative stress-related mechanisms remain speculative and should be evaluated directly in future studies. This interpretation aligns with the observation that NaBu effects were restricted to cryopreserved samples, where oxidative stress is a major limiting factor.

### 4.4. Inter-Individual Variability Suggests Differential Responsiveness to NaBu

A prominent feature of the present study is the substantial variability in acetylation responses among individual boars. Similar variability has been widely reported in boar fertility and semen quality. Lucca et al. (2021) demonstrated that differences in progressive motility between boars are strongly associated with fertility outcomes [20]. In addition, Blagojević et al. (2024) showed that nutritional and metabolic factors can influence semen quality and epigenetic-related gene expression [21]. In this context, the observed heterogeneity in acetylation responses may reflect differences in baseline metabolic or epigenetic states between boars. Some individuals may be more responsive to NaBu due to differences in HDAC activity, mitochondrial efficiency, or redox balance. These findings emphasize the importance of considering individual variability when evaluating semen additives and suggest that NaBu supplementation may be more effective in specific subpopulations of boars.

### 4.5. Cryopreservation Remains the Dominant Factor Shaping Sperm Functional Profiles

Principal component analysis visually indicated separation between fresh and frozen–thawed samples, consistent with cryopreservation being a major source of variation in sperm functional profiles. This observation is consistent with previous studies demonstrating that freezing–thawing procedures induce profound alterations in sperm physiology. Jovičić et al. (2020) reported that cryopreservation affects membrane structure, enzyme activity, and energy metabolism [15], while Yeste et al. (2017) emphasized the role of oxidative stress and metabolic disruption in this process [22]. Against this background, the effects of NaBu appear to be secondary and modulatory rather than dominant. While NaBu was associated with changes in selected parameters, such as motility, it did not overcome the overall impact of cryopreservation on sperm physiology. This supports the conclusion that NaBu acts as a supportive factor that may enhance specific aspects of sperm function within the constraints imposed by cryo-induced damage.

### 4.6. Study Limitations

This study has several limitations. First, no a priori power analysis was performed, and the number of boars was limited; therefore, the statistical power to detect small treatment effects or dose × condition interactions may be restricted. Second, although repeated ejaculates were analyzed and the hierarchical structure of the data was accounted for using mixed-effects models, the findings should be interpreted cautiously and validated in larger populations of boars. Third, PCA was used only as an exploratory visualization tool and should not be interpreted as proof of a biological effect. Finally, global acetylation was assessed by Western blotting, which does not exclude changes in specific acetylated proteins or other molecular pathways that may be involved in the response to NaBu. In addition, oxidative stress and metabolic parameters, including ROS levels, ATP content, lipid peroxidation, antioxidant status, and HDAC activity, were not assessed. Therefore, the biological mechanisms underlying the observed motility-related changes remain unresolved and should be investigated in future studies using targeted metabolic, oxidative stress, and acetyl-proteomic approaches.

## 5. Conclusions

This study showed that sodium butyrate was associated with changes in selected in vitro functional parameters of frozen–thawed boar spermatozoa, particularly motility-related parameters, whereas no corresponding significant effects were observed in fresh semen after 24 h storage. These functional changes were not associated with detectable statistically significant changes in global protein acetylation under the conditions tested.

The findings suggest that the response to NaBu may involve mechanisms other than broad changes in global protein acetylation, potentially including specific protein acetylation events, metabolic pathways, mitochondrial function, or stress-response mechanisms. However, these mechanisms require further investigation using targeted molecular and functional approaches.

Due to the limited number of boars and the absence of fertility-related outcomes, the present findings should be interpreted as preliminary in vitro evidence. Because fertility-related outcomes were not assessed, improved post-thaw motility should not be interpreted as direct evidence of improved fertilizing capacity. Further studies with larger animal cohorts are needed before NaBu can be recommended as an additive for boar semen cryopreservation.

## Figures and Tables

**Table 1:** Effect of sodium butyrate (NaBu; 0–1 mM) on selected functional parameters of frozen–thawed boar sperm. Data are presented as mean ± SD. Statistical analysis was performed using linear mixed-effects models with dose as a fixed effect and boar and collection nested within boar as random effects. Pairwise comparisons between doses were conducted using estimated marginal means with Tukey adjustment. Different superscript letters within a row indicate significant differences between treatment groups based on Tukey-adjusted post hoc comparisons (*p* < 0.05). The complete statistical output, including adjusted *p*-values, effect estimates, and 95% confidence intervals, is provided in Supplementary Table S2.

Parameter	0 mM	0.5 mM	0.75 mM	1 mM
Total motility (%)	82.79 ± 4.53 ^b^	85.59 ± 3.10 ^a^	81.94 ± 2.67 ^b^	85.71 ± 3.46 ^a^
Progressive motility (%)	53.62 ± 5.44 ^b^	59.78 ± 4.25 ^a^	56.03 ± 3.41 ^ab^	58.24 ± 6.15 ^a^
Mitochondrial potential—high (%)	81.47 ± 4.56 ^ab^	84.23 ± 2.81 ^a^	80.38 ± 2.91 ^b^	83.62 ± 4.94 ^a^
Mitochondrial potential—low (%)	18.53 ± 4.56 ^ab^	15.77 ± 2.81 ^b^	19.62 ± 2.91 ^a^	16.38 ± 4.94 ^b^

**Table 2:** Log10-transformed acetylation levels in boar sperm treated with sodium butyrate (NaBu; 0–1 mM) under fresh after 24 h storage and frozen–thawed conditions. Acetylation values were log10-transformed prior to statistical analysis. Statistical analysis was performed using linear mixed-effects models followed by Tukey’s post hoc test with Benjamini–Hochberg correction for multiple comparisons. For clarity, only comparisons versus control (0 mM) are shown. No statistically significant differences were observed between NaBu doses in either condition (all adjusted *p*-values > 0.05).

Condition	Dose (mM)	log10 Acetylation (Mean ± SD)	Comparison (Tukey)	Adjusted *p*-Value
Fresh after 24 h storage	0	7.705 ± 0.26	–	–
	0.5	8.173 ± 0.65	0 vs. 0.5	0.977
	0.75	8.018 ± 0.55	0 vs. 0.75	1.000
	1	7.359 ± 0.23	0 vs. 1	0.790
Frozen–thawed	0	7.778 ± 0.14	–	–
	0.5	7.825 ± 0.11	0 vs. 0.5	0.968
	0.75	7.800 ± 0.10	0 vs. 0.75	0.994
	1	7.930 ± 0.18	0 vs. 1	0.699

## Data Availability

The data presented in this study are available on request from the corresponding author due to restrictions.

## Supplementary Materials

The following supporting information can be downloaded at: https://www.mdpi.com/article/10.3390/ani16131952/s1.
