# Supplementary material for: Effects of Sodium Butyrate on Sperm Function and Protein Acetylation in Fresh and Frozen–Thawed Boar Spermatozoa
Source: Animals (Basel). 2026 Jun 24;16(13):1952. doi: 10.3390/ani16131952 (PMC13360045; doi:10.3390/ani16131952)
Supplement: Supplementary file 1 [file animals-16-01952-s001.zip › animals-4350143-supplementary Figure S1.pdf]

Line 1 - 1mM boar 1
Line 2 - 0mM boar 2
Line 3- 0,5mM boar 2
Line 4- 0,75mM boar 2
Line 5- 1mM boar 2
Line 6- 0mM boar 3
Line 7- 0,5mM boar 3
Line 8-0,75mM boar 3
Line 9- 1mM boar 3
Line 10- Marker

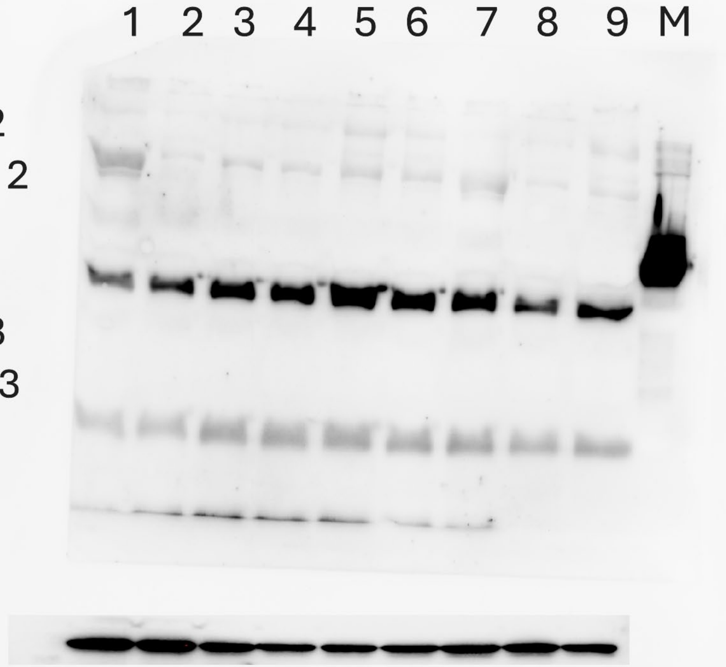

Beta-Tubulin
